# An algorithmic approach to soft-tissue reconstruction around the knee using anterolateral thigh perforator flap in patients with post-traumatic knee osteomyelitis

**DOI:** 10.3389/fsurg.2023.982669

**Published:** 2023-02-06

**Authors:** Seong-Ho Jeong, Seung-Hee Baik, Sik Namgoong, Eun-Sang Dhong, Seung-Kyu Han

**Affiliations:** Department of Plastic Surgery, Korea University Guro Hospital, Seoul, South Korea

**Keywords:** algorithmic approach, knee soft-tissue defects, anterolateral thigh perforator flap, post-traumatic chronic osteomyelitis, radical debridement of osteomyelitis

## Abstract

**Background:**

Free tissue transfer to the knee region in patients with chronic post-traumatic knee osteomyelitis (CTKOM) poses a great challenge to surgeons because the remaining soft tissues adjacent to defects, including vascular structures, are usually damaged by chronic inflammation and multiple debridements. Thus, we developed an algorithm to help select the optimal recipient vessels and appropriate anterolateral thigh perforator (ALTP) flap type. In addition, we performed surgery using this algorithm and achieved successful reconstructions. This study aims to review our experiences in algorithmic reconstruction and assess its efficacy.

**Methods:**

According to the defect size and location, our algorithm suggested the use of various-shaped ALTP flaps with centrally located perforators (Cen-ALTP flap) or eccentrically located perforators (Ecc-ALTP flap). Besides, through the algorithm, one recipient vessel was selected among three candidates, including descending branch of the lateral circumflex femoral artery (DB-LCFA), anterior tibial artery (ATA), and posterior tibial artery (PTA). Based on this algorithmic decision, we performed individualized soft tissue reconstructions of the knee in 21 patients with CTKOM, between March 2013 and June 2021. The medical records of the patients were retrospectively reviewed.

**Results:**

The Cen-ALTP flap (*n* = 15) and ATA (*n* = 9) were the most commonly used for reconstruction. The Cen-ALTP flap anastomosed to the ATA was most commonly selected (*n* = 7) using the algorithm, followed by the Cen-ALTP flap anastomosed to the DB-LCFA (*n* = 5), and the Cen-ALTP flap anastomosed to the PTA (*n* = 3). All transferred ALTP flaps survived the follow-up period. Postoperative venous congestion in two patients and hematoma in one patient were resolved by immediate treatment. The postoperative course was uneventful.

**Conclusion:**

During free ALTP flap transfer to CTKOM-related knee defects, we could select the optimal recipient vessel and appropriate flap type using our algorithm and obtain excellent reconstructive outcomes. Therefore, we believe that our algorithm could provide helpful guidance to reconstructive surgeons on free ALTP flap transfer to reconstruct CTKOM-related soft tissue defects.

## 1. Introduction

The lower extremities are more frequently affected by chronic post-traumatic osteomyelitis compared to other body parts (1). Therefore, surgeons often encounter patients with osteomyelitis that occurs in the knee area following traumatic injuries. Osteomyelitis is a slowly progressing disease with periods of deterioration followed by improvement and a hard-to-cure infection with a high recurrence rate (2). Therefore, repetitive radical debridement and drainage procedures are usually required. However, therapeutic approaches focusing on bony infections can unintentionally damage soft tissues and lead to wound breakdown and local scar contracture (3). In addition, a prolonged inflammatory state in soft tissue wounds affected by bony osteomyelitis can cause soft tissue hardening and loss of elasticity, which may hinder wound healing. Therefore, the replacement of damaged soft tissues with healthy ones is commonly required. This reconstructive procedure can facilitate the cure of osteomyelitis by increasing the delivery of antibiotics and providing stable coverage enough to endure repeated surgeries (4).

Although various reconstructive methods, including local skin or muscle flaps, distant flaps, and free tissue transfer, have been used to restore soft tissue coverage of the knee joints (5, 6), there is still no universally accepted standard method because the available reconstructive options are limited depending on the extent of the soft tissue damage in each patient (7). Nevertheless, free flap coverage is known as an ideal option to replace extensive and severely damaged soft tissues around knee because it can provide well-perfused tissue from outside the zone of injury without additional surgical trauma to an already injured knee area (8). Although it requires a high level of technical proficiency and infrastructure (5), previous studies have reported that free tissue transfer in the peri-knee region is a reliable procedure, with a higher success rate of over 90% (9). Free flaps can be transferred successfully by a well-trained surgeon if the recipient vessels are appropriately selected and prepared (10).

**Figure 1:**
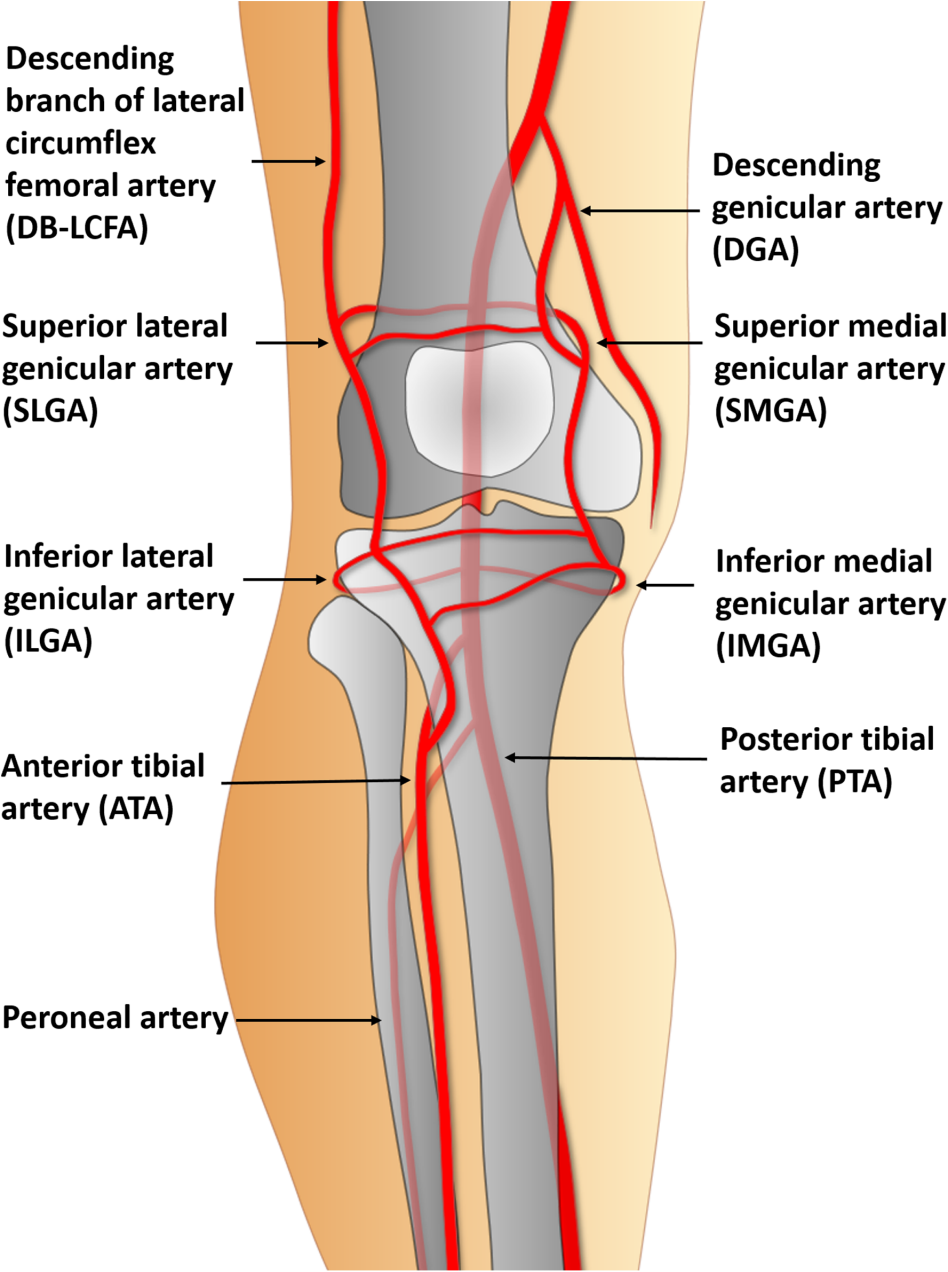
Potential recipient arteries in the knee region for free tissue transfer.

**Figure 2:**
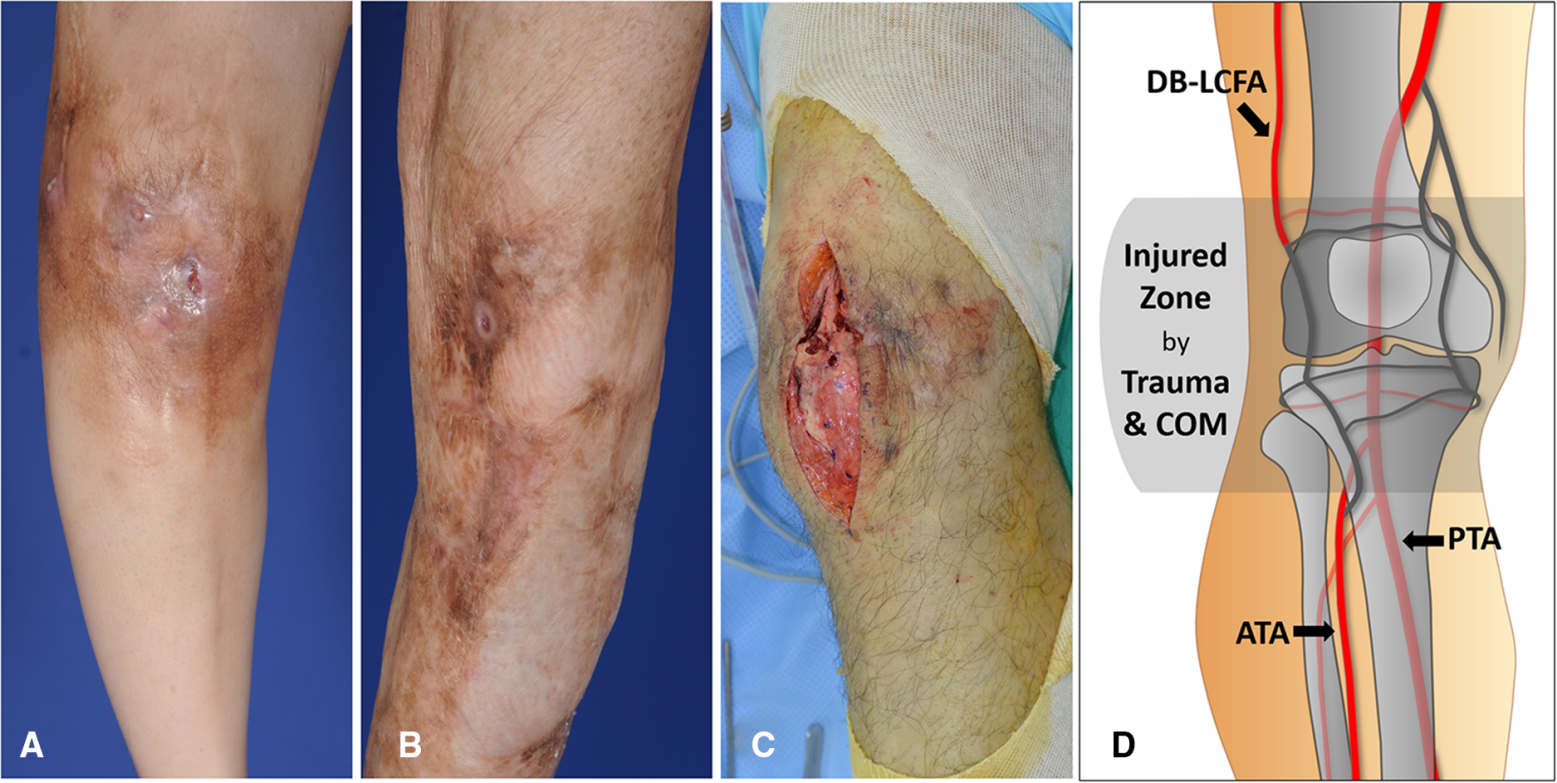
Damaged soft tissues around the knee caused by repetitive treatment of chronic posttraumatic osteomyelitis (COM) (**A**) A 49-year-old woman had a deteriorating wound around the knee accompanied by pus drainage due to osteomyelitis. The skin discoloration and thinning resulting from chronic inflammation are observed. (**B**) A 43-year-old man showed improvement in wound status without any discharge. Extensive scar formation following multiple debridement of COM is identified around the knee. (**C**) Destructed soft tissues mixed with scars was exposed during debridement of COM in a 39-year-old man. (**D**) Most of the arteries in the genicular arterial network are possibly damaged and unusable as recipient vessels because they are likely to be traumatized by initial trauma and COM. On the contrary, descending branch of lateral circumflex femoral artery (DB-LCFA), anterior tibial artery (ATA), and posterior tibial artery (PTA) tend to be safe from the traumatic damage due their deep location outside the injured zone and protection by muscles.

Recipient vessel selection is the most complex and challenging aspect of free tissue transfer in knee lesions. Almost every vessel near the knee could be used as a recipient vessel (11) (Figure 1). However, the location and severity of the initial bone and soft tissue trauma, previous multiple surgeries, and chronic inflammation due to osteomyelitis might change the typical vascular anatomy. Consequently, traditional recipient vessels are not always available (12). Thus, the descending branch of the lateral circumflex femoral artery (DB-LCFA) and anterior or posterior tibial artery (ATA or PTA) and their concomitant veins are comparatively better options for recipient vessels because they are well preserved by covering muscles and are located far from the injured area (Figure 2). However, using these vessels as recipient vessels is complicated because they are located far from the defect and are set deep in the muscles. Therefore, to achieve successful free flap transfer based on these vessels, a versatile flap that can cope with various demands of pedicle length and flap size is required.

Recently, the anterolateral thigh perforator flap (ALTP flap) has been widely adopted to reconstruct serious soft tissue defects with deep infection because many studies have shown that its infection control abilities are similar to muscle flaps (13). In addition, the ALTP flap is versatile, with a long pedicle and skin paddles of various sizes. Therefore, we tried to replace the damaged peri-knee soft tissues with an ALTP-free flap anastomosed to the DB-LCFA, ATA, PTA, and their concomitant veins. Additionally, based on our earlier experience, we developed an algorithm to help select the optimal recipient vessels and appropriate flap design according to the defect size and location. Thereafter we performed free ALTP flap transfer to reconstruct osteomyelitis-related knee defects using the recipient vessel and specific flap shape selected through our algorithm.

In this article, we aim to provide delicate reconstructive strategies for recovering healthy soft tissue coverage of the injured knee area affected by osteomyelitis, considering individual anatomical features of donor and recipient sites. In addition, we describe our detailed surgical procedure according to the algorithm developed from these strategies and present the post-operative outcomes.

## 2. Patients and methods

Free ALTP flaps were used to reconstruct osteomyelitis-related soft tissue defects around the knee at the Korea University Guro Hospital between March 2013 and June 2021. The surgical procedures were consistent with the ethical guidelines of the 1975 Declaration of Helsinki. Twenty-one soft tissue defects in 21 patients (17 men and four women) were reconstructed using ALTP flaps (Table 1). The patients' age ranged from 24 to 70 years, with an average of 40.6 years. All patients had persistent post-traumatic osteomyelitis of the knee area diagnosed by magnetic resonance imaging, bone scan, and bone tissue culture. The mean interval from the initial trauma to the definite reconstruction was 46 months (range: 22–115), and the mean interval from the diagnosis to the definite reconstruction was 23 months (range: 9–71).

**Table 1:** Summary of patients’ information and surgical outcomes.

Case No	Sex/Age (years)	Defect location^a^	Defect size (cm)	Flap size (cm)	Flap type	Perf. no.	Recipient vessel^b^	Donor site (Thigh)	Donor closure	Surgical outcome	Complications	Option No^c^
1	M/35	Supra-lateral	17 × 11	20 × 12	Cen-ALTP	One	DB-LCFA (2)	Contralateral	Skin graft	Survival	None	1
2	M/46	Infra-medial	11 × 7	13 × 10	Cen-ALTP	One	PTA (2)	Contralateral	1′ repair	Survival	None	5
3	F/49	Infra-lateral	13 × 11	15 × 12	Cen-ALTP	One	ATA (1)	Contralateral	1’ repair	Survival	None	4
4	M/67	Supra-medial	17 × 10	22 × 13	Ecc-ALTP	Two	ATA (2)	Contralateral	Skin graft	Survival	Hematoma	2
5	M/24	Supra-central	14 × 12	16 × 14	Cen-ALTP	One	DB-LCFA (2)	Contralateral	Skin graft	Survival	None	1
6	M/50	Infra-lateral	19 × 6	21 × 10	Cen-ALTP	Two	ATA (2)	Contralateral	Skin graft	Survival	None	4
7	M/40	Infra-medial	12 × 8	13 × 11	Cen-ALTP	One	PTA(1), SV (1)	Ipsilateral	1’ repair	Survival	None	5
8	M/70	Infra-lateral	14 × 11	16 × 12	Cen-ALTP	One	ATA (2)	Contralateral	Skin graft	Survival	None	4
9	M/35	Infra-medial	17 × 11	20 × 12	Cen-ALTP	Two	ATA (2)	Contralateral	Skin graft	Survival	None	4
10	M/46	Supra-lateral	14 × 11	16 × 15	Cen-ALTP	One	DB-LCFA (2)	Contralateral	1’ repair	Survival	VC	1
11	M/50	Supra-lateral	13 × 7	20 × 10	Ecc-ALTP	One	PTA (2), VG	Contralateral	1’ repair	Survival	None	3
12	M/67	Infra-lateral	17 × 9	19 × 11	Cen-ALTP	Two	PTA (1), VG	Contralateral	Skin graft	Survival	None	5
13	M/24	Infra-lateral	18 × 7	21 × 11	Ecc-ALTP	One	DB-LCFA (2)	Ipsilateral	Skin graft	Survival	None	7
14	F/49	Infra-medial	13 × 10	16 × 12	Cen-ALTP	One	ATA (1), SV (1)	Contralateral	Skin graft	Survival	None	4
15	M/40	Infra-lateral	17 × 10	20 × 12	Cen-ALTP	Two	ATA (2)	Contralateral	Skin graft	Survival	None	4
16	M/70	Infra-medial	12 × 7	19 × 10	Ecc-ALTP	Two	DB-LCFA (2)	Contrallateral	Skin graft	Survival	None	6
17	F/58	Supra-lateral	18 × 11	21 × 11	Cen-ALTP	Two	DB-LCFA (2)	Contrallateral	Skin graft	Survival	None	1
18	M/44	Infra-lateral	18 × 8	20 × 12	Ecc-ALTP	Two	DB-LCFA (2)	Contrallateral	Skin graft	Survival	None	7
19	M/56	Supra-lateral	18 × 11	20 × 12	Ecc-ALTP	One	ATA (2)	Contrallateral	Skin graft	Survival	VC	2
20	M/39	Infra-lateral	14 × 12	15 × 13	Cen-ALTP	One	ATA (2)	Contrallateral	1’ repair	Survival	None	4
21	F/61	Supra-lateral	13 × 13	15 × 13	Cen-ALTP	One	DB-LCFA (2)	Contrallateral	1’ repair	Survival	None	1

No, number; Perf., perforator; ALTP, anterolateral thigh perforator; Cen-ALTP flap, ALTP flap with centrally located perforators; DB-LCFA, descending branch of lateral circumflex iliac artery and its concomitant veins; PTA, posterior tibial artery and its concomitant veins; ATA, anterior tibial artery and its concomitant veins; Ecc-ALTP flap, ALTP flap with eccentrically located perforators; SV, superficial vein; VG, vein graft; VC, venous congestion.

^a^ Relative location of the center of each defect based on a horizontal line through the center of the patella bone.

^b^ The numbers in parenthesis refer to the number of venous anastomoses using concomitant veins.

^c^ Option number on the algorithm.

Chronic osteomyelitis is characterized by periods of fluctuating disease activity. With the aggravation of the disease, the patients suffered from severe bone and soft tissue infections, which required long-term intravenous antibiotic therapy, repeated surgical debridement and irrigation, and frequent wound dressing changes. All patients enrolled in this study had undertaken repeated surgical debridement 15 times on average (range: 7–30) and had unhealthy soft-tissue conditions, usually presented as rigid and hyperpigmented skin due to chronic inflammatory damage. However, they had not undertaken any local or free flap procedures. Finally, the thorough physical examination revealed that local reconstructive options were inappropriate for soft tissue coverage for the knee region because of poor vascularity.To improve the cure rate in these patients, after wide debridement of almost all bone and soft tissues affected by infection, definite reconstruction with highly vascularized healthy soft tissue is required. Therefore, free transfer of ALTP flaps with sufficient soft tissues was performed for all patients. All soft tissue defects described in this study resulted from radical debridement of the necrotic and infected tissues. In 13 defects, the defect center was located below the patellar center. In contrast, in eight defects, the center was above it. The sizes of the defects ranged from 11 × 7 (77 cm^2^) to 18 × 11 (198 cm^2^) (mean, 147 cm^2^). We performed a detailed retrospective review of the medical records of all the patients.

### 2.1. Algorithm

**Figure 3:**
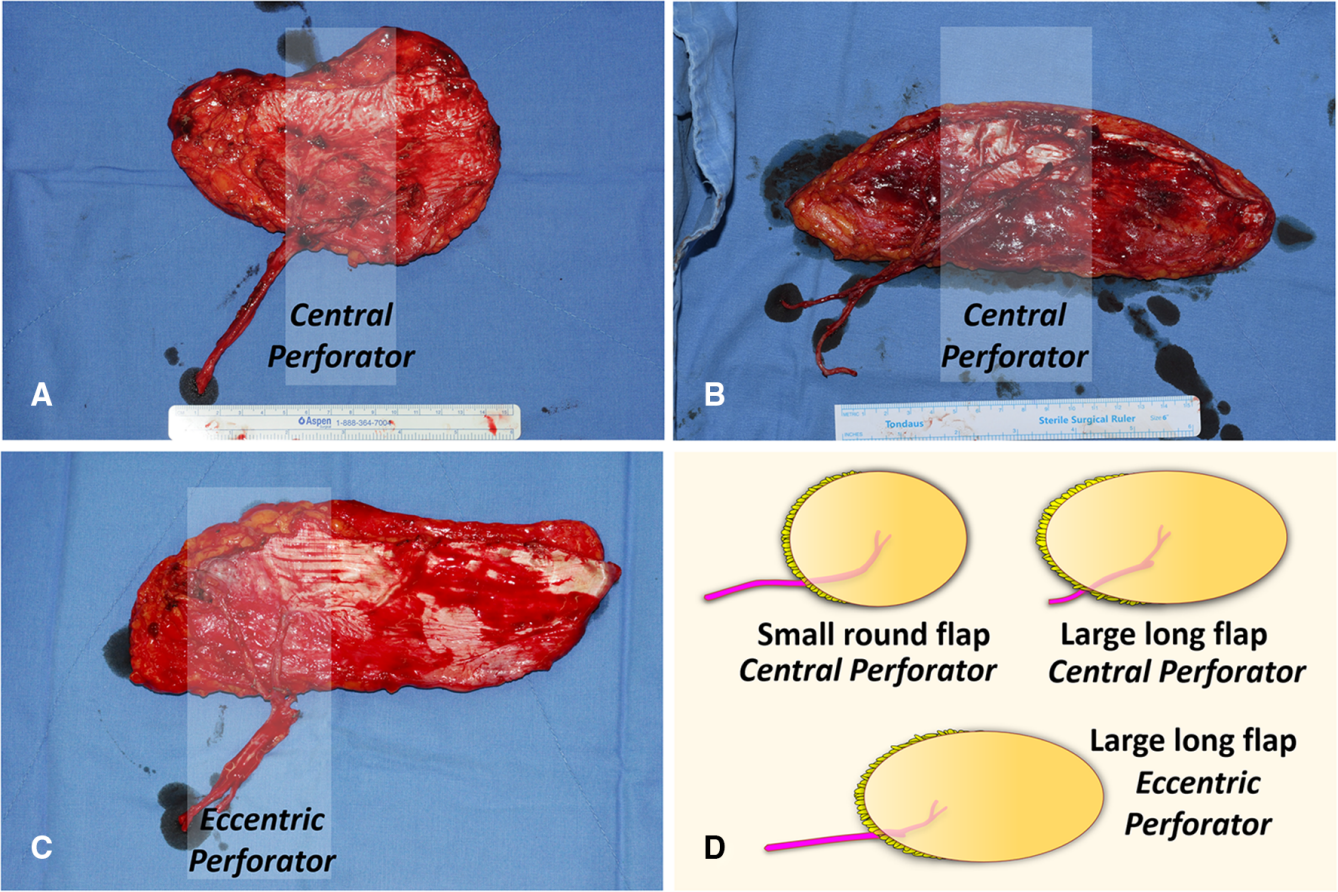
Anterolateral thigh perforator (ALTP) flaps with various shapes and sizes used for soft tissue reconstruction around the knee. (**A**) ALTP flap with small round skin paddle supplied by perforators located in the central area of the flap (Cen-ALTP flap). (**B**) ALTP flap with large long skin paddle supplied by perforators located in the central area of the flap (Cen-ALTP flap). (**C**) ALTP flap with large long skin paddle supplied by perforators located eccentrically in the flap (Ecc-ALTP flap). (**D**) Cen-ALTP flap is first considered for use due to their stable blood supply. As the defect size increases, an ALTP flap is required in the order of Cen-ALTP flap with small round skin paddle, Cen-ALTP flap with large long skin paddle, and Ecc-ALTP flap with large long skin paddle.

We used two different types of ALTP flaps. One is the ALTP flap with centrally located perforators (Cen-ALTP flap), and the other is the ALTP flap with eccentrically located perforators (Ecc-ALTP flap). We preferred to use the Cen-ALTP flap due to its stable blood supply to all areas of the flap. However, sometimes, to reconstruct a defect that is located far from the anastomotic point of the flap pedicle or to cover an extensive defect, the Ecc-ALTP flap was used alternatively. Depending on the defect size, the Cen-ALTP flap can have various shapes, from a small round shape (Figure 3A) to a large long shape (Figure 3B), whereas the Ecc-ALTP flap has only a large long shape to reach the area far from the provisional vascular anastomosis site (Figure 3C).

**Figure 4:**
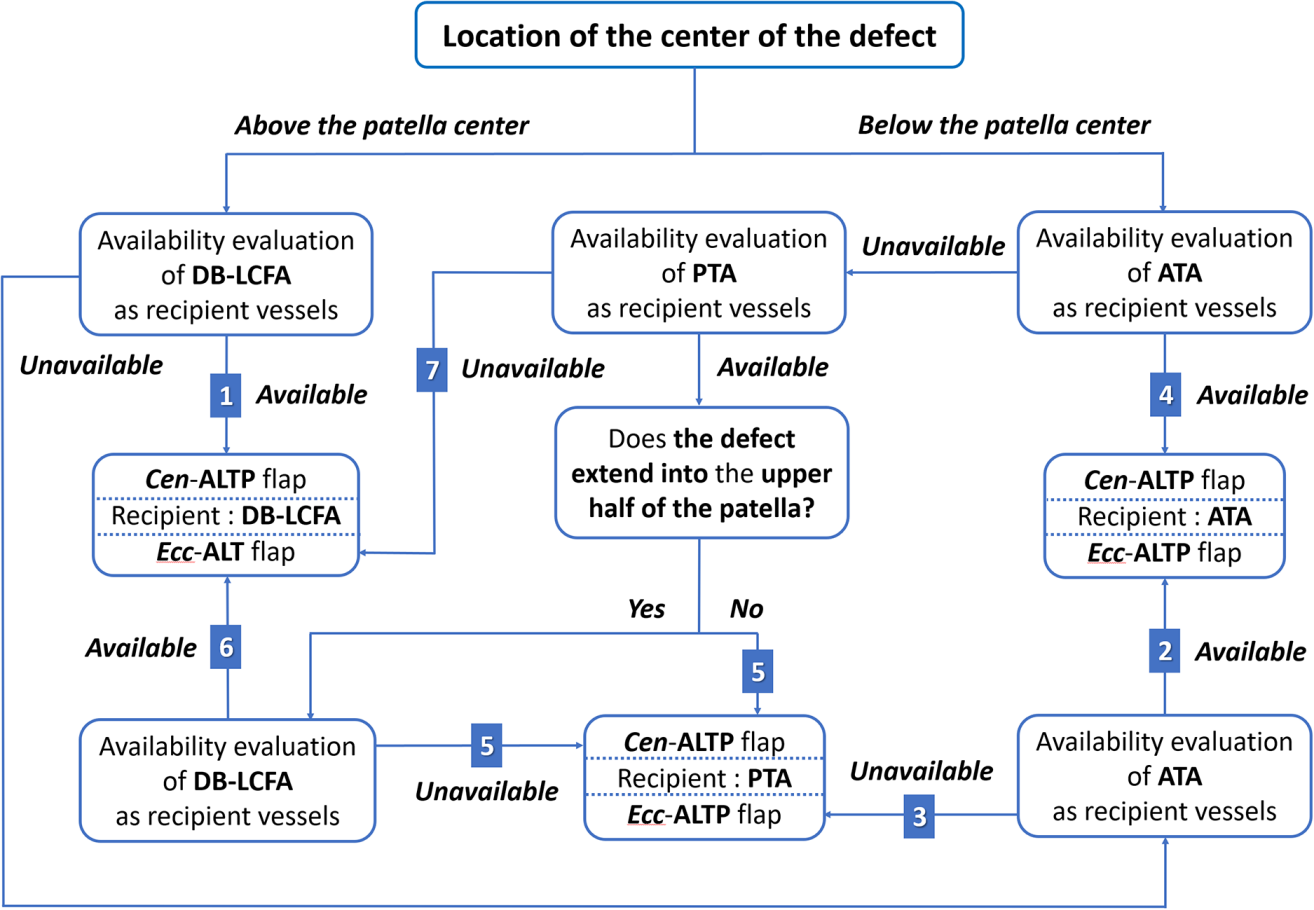
An algorithmic approach to selecting the optimal ALTP flap-type and most appropriate recipient vessels for the soft-tissue reconstruction of the knee area in patients with chronic post-traumatic osteomyelitis. The available options on the combination of the flap-type and the recipient vessels were given a number from 1 to 7. (DB-LCFA, descending branch of the lateral circumflex femoral artery; PTA, posterior tibial artery; ATA, anterior tibial artery; ALTP, anterolateral thigh perforator; Cen-ALTP flap, ALTP flap with centrally located perforators; Ecc-ALTP, ALTP flap with eccentrically located perforators).

wFurthermore, we made every effort to use only healthy recipient vessels that were well-preserved from osteomyelitis and initial trauma to avoid free flap failure that might lead to limb loss. Thus, we excluded genicular arteries vulnerable to trauma and decided to use only three vessels: DB-LCFA, ATA, and PTA. Consequently, we established a strategic algorithm to select the optimal flap type and the most appropriate recipient vessels for free ALTP flap transfer according to the location of the defect (Figure 4).

#### 2.1.1. Soft tissue defects above the patella

**Figure 5:**
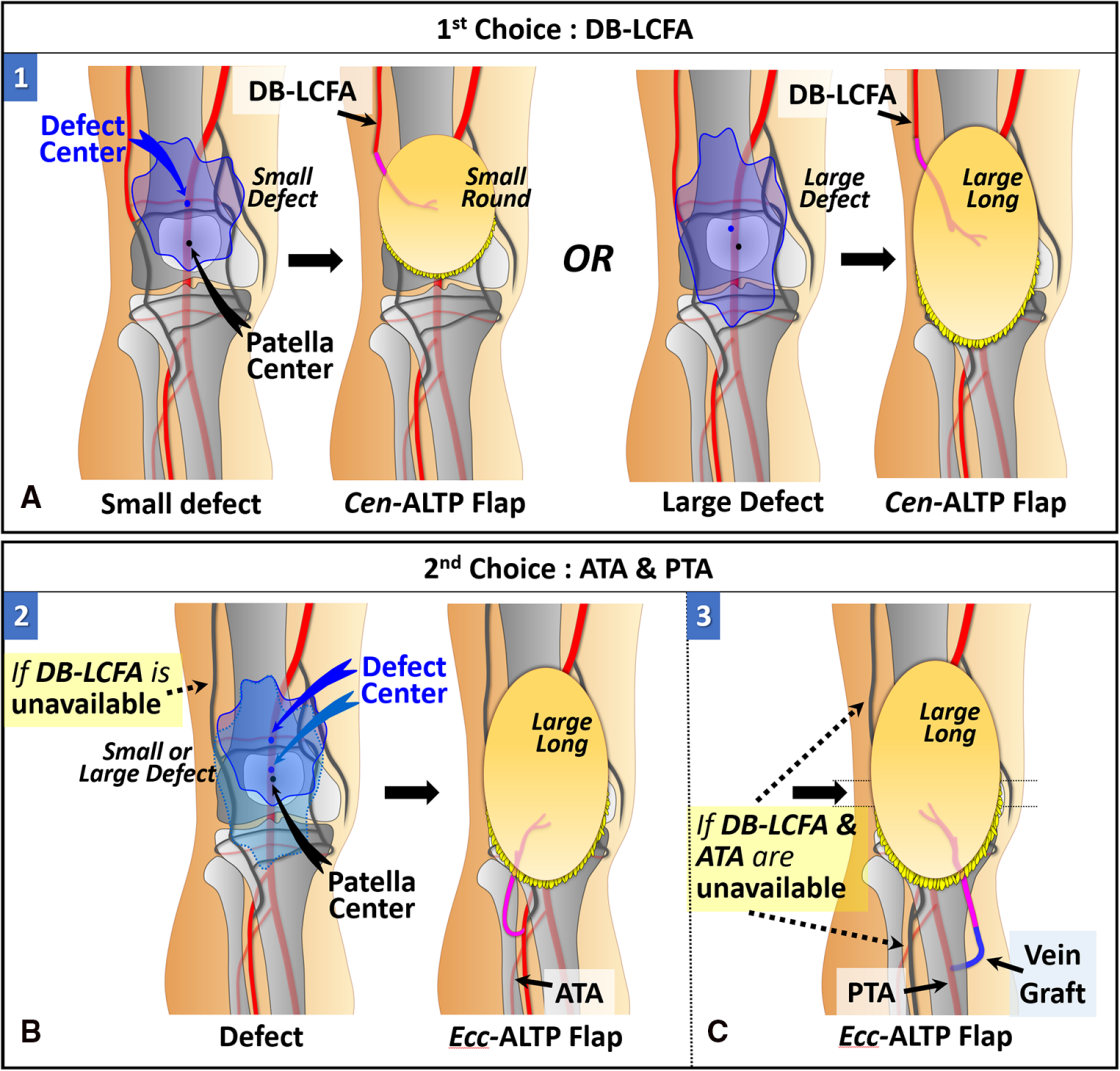
Algorithmic options for soft tissue defects above the patella in which the defect center is located above the patella center. (The number in each figure piece indicates the corresponding option number in the algorithm) (**A**) DB-LCFA is the first choice as a recipient vessel because of its proximity to the defect and easy accessibility. Depending on the defect size, Cen-ALTP flap with a small round or large long skin paddle can be used for free tissue transfer. (**B**) If the DB-LCFA is unavailable to use, ATA (1st choice) or PTA (2nd choice) could be used as a recipient vessel irrespective of the defect's size. Firstly, If only DB-LCFA is unavailable as a recipient vessel, Ecc-ALTP flap with a large long skin paddle, which is anastomosed to ATA, is used for free tissue transfer. (**C**) Secondly, if both DB-LCFA and ATA are unavailable as recipient vessels, Ecc-ALTP flap with a large long skin paddle is transferred using PTA accompanied by a vein graft. (DB-LCFA, descending branch of the lateral circumflex femoral artery; ALTP flap, anterolateral thigh perforator flap; Cen-ALTP flap, ALTP flap with centrally located perforators; ATA, anterior tibial artery; PTA, posterior tibial artery; Ecc-ALTP flap, ALTP flap with eccentrically located perforators).

When the defect center is located above the patellar center, DB-LCFA is the first choice as a recipient vessel because of its proximity to the defect and easy accessibility by extending the incision from the defect. The Cen-ALTP flap can be used for free tissue transfer. In addition, depending on the defect size, the various flap shape, from small round to large long, can be used to achieve successful coverage of the defect. (Figure 5A). If DB-LCFA is unavailable, ATA is considered as the second-best altenative for a recipient vessel. In this case, the Ecc-ALTP flap with a large long skin paddle is employed because the ATA was far away from the superior border of the defect (Figure 5B). If both the DB-LCFA and ATA are unavailable, PTA can be selected as a last resort with the Ecc-ALTP flap. When PTA is used as a recipient vessel for the free transfer of a large Ecc-ALTP flap, a vein graft is commonly required to allow tension-free vascular anastomosis because PTA is in the deep posterior compartment of the lower leg, and the large skin paddle of the flap could limit the mobility of the flap pedicle during flap insetting (Figure 5C).

#### 2.1.2. Soft tissue defects below the patella

**Figure 6:**
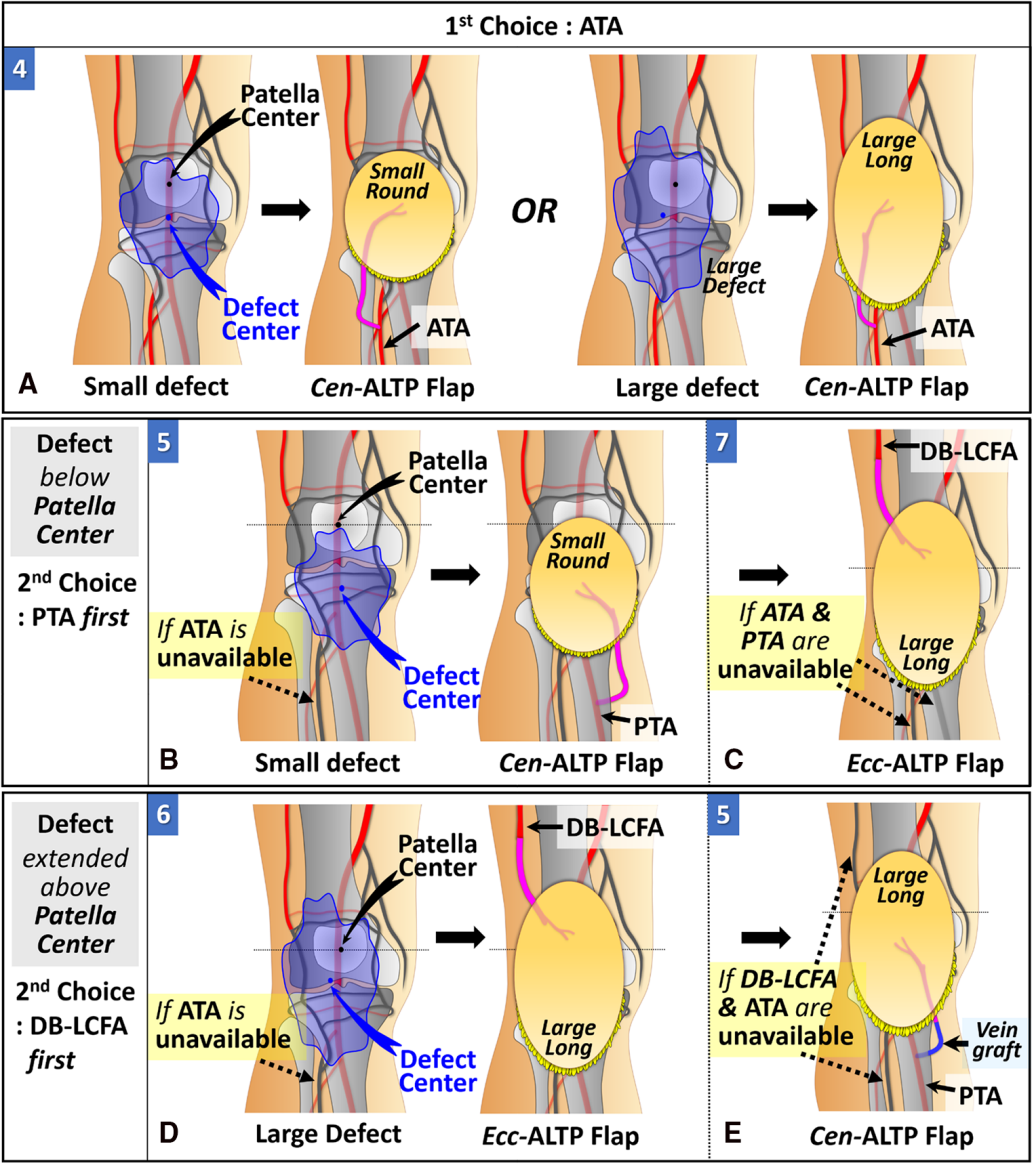
Algorithmic options for soft tissue defects below the patella in which the defect center is located below the patella center. (The number in each figure piece indicates the corresponding option number in the algorithm) (**A**) ATA is the first choice as a recipient vessel because of its proximity to the defect and easy accessibility. Depending on the defect size, Cen-ALTP flap with a small round or large long skin paddle can be used for free tissue transfer. (**B**) if the ATA is unavailable to use and the whole defect is located below the patella center, PTA (1st choice) or DB-LCFA (2nd choice) could be used as a recipient vessel. Firstly, if only ATA is unavailable as a recipient vessel, Cen-ALTP flap with small round skin paddle, which is anastomosed to PTA, is used for free tissue transfer. (**C**) Secondly, if both ATA and PTA are unavailable as recipient vessels, Ecc-ALTP flap with large long skin paddle, which is anastomosed to DB-LCFA is used. (**D**) If the ATA is unavailable to use and the defect extends into the upper half of the patella, DB-LCFA (1st choice) or PTA (2nd choice) could be used as a recipient vessel. Firstly, if only ATA is unavailable as a recipient vessel, Ecc-ALTP flap with large long skin paddle, which is anastomosed to DB-LCFA, is used for free tissue transfer. (**E**) Secondly, if both ATA and DB-LCFA are unavailable as recipient vessels, Cen-ALTP flap with large long skin paddle is transferred using PTA accompanied by a vein graft. (ATA, anterior tibial artery; ALTP flap, anterolateral thigh perforator flap; Cen-ALTP flap, ALTP flap with centrally located perforators; PTA, posterior tibial artery; DB-LCFA, descending branch of the lateral circumflex femoral artery; Ecc-ALTP flap, ALTP flap with eccentrically located perforators).

When the defect center is located below the patella center, ATA is the first choice as a recipient vessel for the same reason as we explained for DB-LCFA above. The Cen-ALTP flap can be used for free tissue transfer. The Cen-ALTP flap can be used for free tissue transfer. In addition, depending on the defect size, the flap shape can be optimized to obtain ample coverage of the defect (Figure 6A). If ATA is unavailable for use as a recipient vessel, an alternative vessel is selected depending on the size of the defect. For defects with the whole area located below the patella center (small defect), PTA is selected as the recipient vessel because of its proximity. Next, the Cen-ALTP free flap with a small round skin paddle, which is anastomosed to this vessel, was employed without a vein graft (Figure 6B). However, if PTA is unavailable, they should be covered by a large Ecc-ALTP free flap using DB-LCFA as a recipient vessel despite their small size (Figure 6C). For defects with their superior portion extended to the upper half of the patella (large defect), although PTA is closer to the defects than DB-LCFA, DB-LCFA is preferentially used as a recipient vessel to avoid vein grafts (Figure 6D). Next, the Ecc-ALTP free flap anastomosed to this vessel was adopted to cover the relatively large defects. In cases where DB-LCFA was also unavailable, the Cen-ALTP flap with the pedicle anastomosed to PTA can finally be used. However, in these cases, a vein graft is commonly required to obtain tension-free pedicle anastomosis (Figure 6E).

### 2.2. Surgical technique

All ALTP flaps were harvested in the traditional manner. While the orthopedic surgeons debride the bone and soft tissues affected by osteomyelitis, plastic surgeons harvested the ALTP flap simultaneously. To facilitate the two-team approach, the ALTP flap was usually harvested from the contralateral thigh except in some cases where it was damaged by previous surgeries or traumas. Before surgery, the recipient vessels were evaluated by computed tomographic angiography and venography. The perforators supplying the lateral thigh were detected using a handheld Doppler and marked. First, we designed the flap on the lateral thigh in an appropriated shape according to the defect size, with perforators located centrally or eccentrically. After identifying the perforators *via* the medial incision, the main pedicle (usually the DB-LCFA) was dissected proximally to its origin from the LCFA, and the whole flap was harvested. We usually included only one or two sizable perforators in each ALTP flap. Even when we harvested ALTP flaps with large skin paddles, we just selected one or two perforators, which were located centrally or eccentrically, because these perforators alone can provide sufficient blood flow to most ALTP flaps. Furthermore, if the large ALT flap is harvested with multiple perforators distributed in the whole flap, these perforators are likely to preclude tension-free pedicle anastomosis or flap insetting by limiting the motion of the pedicle and skin paddle. Finally, the flap was transferred to the defect around the knee and the pedicle was anastomosed to the predetermined recipient vessel.

## 3. Results

The harvested ALTP flap size ranged from 13 × 10 cm^2^ (130 cm^2^) to 22 × 13 (286 cm^2^; mean, 212 cm^2^). ALTP flaps based on central perforators in 15 patients and eccentric perforators in six patients were transferred. ATA was the most commonly utilized recipient vessel (*n* = 9), followed by DB-LCFA (*n* = 8) and PTA (*n* = 4). Among the surgical options presented in the algorithm, option number four, which adopted the Cen-ALTP flap, which is anastomosed to the ATA, was the most commonly selected (*n* = 7), followed by option number one (Cen-ALTP flap anastomosed to DB-LCFA; *n* = 5) and option number five (Cen-ALTP flap anastomosed to PTA; *n* = 3). The arterial anastomosis was performed in an end-to-side manner in all patients. In addition, two venous anastomoses to the accompanying veins of the selected artery were performed in an end-to-end manner in most patients (*n* = 17).

All transferred ALTP flaps survived during the follow-up period of 9–72 months (mean 27 months). However, venous congestion occurred in two patients using LCFA and two concomitant veins as recipient vessels. We explored the anastomosed veins as soon as we detected the congestion during close postoperative monitoring. We then reconnected a previously obstructed vein after removing the intravascular thrombus in one patient and switched two venous anastomoses to a single superficial venous anastomosis in the other patient. One patient suffered from hematoma, which resolved without any adverse effects on the flap. Most donor sites were primarily closed when the regular oval-shaped flaps were used (six of nine cases). Conversely, every donor site for the long oval-shaped flaps was covered with a split-thickness skin graft, except for one donor site closed by primary repair.

## 4. Case reports

### 4.1. Case 1 (patient No. 5)

**Figure 7:**
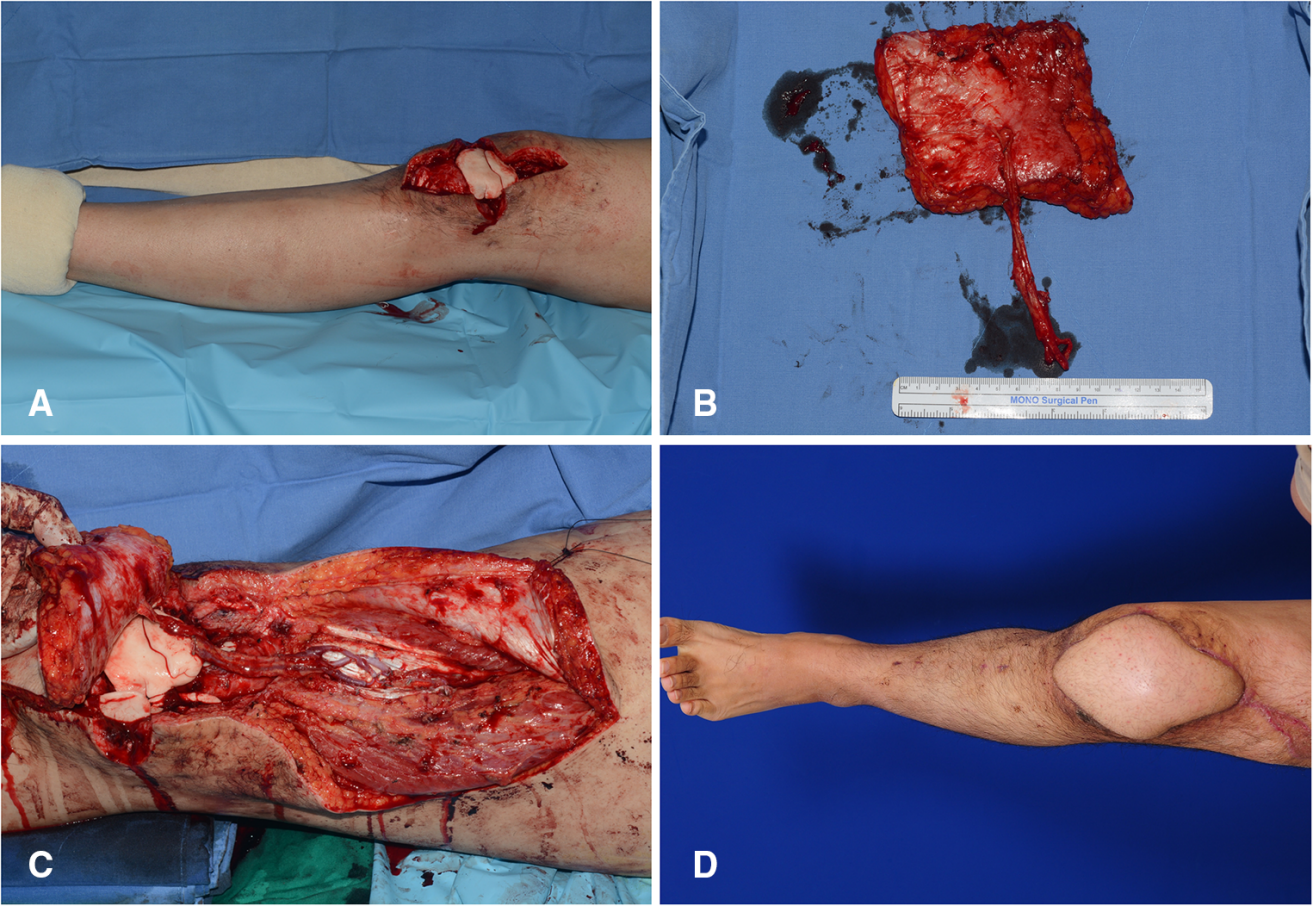
A clinical case showing the reconstruction of the soft-tissue defect around the knee using Cen-ALTP flap, which is anastomosed to DB-LCFA and its concomitant veins. (Case No. 5; Algorithmic option No.1) (**A**) Soft-tissue defect was created after radical debridement of the knee joint in a 24-year-old male patient with chronic osteomyelitis. The defect center was above the patella center. (**B**) Because the defect size was relatively small, Cen-ALTP flap with small round skin paddle (16 × 14 cm2) was harvested. (**C**) The flap pedicle was anastomosed to DB-LCFA and its concomitant veins. (**D**) Result 5 months postoperatively. (Cen-ALTP flap, anterolateral thigh perforator flap with centrally located perforators; DB-LCFA, descending branch of the lateral circumflex femoral artery).

A 24-year-old man suffered from chronic post-traumatic osteomyelitis of the knee joint. After the final debridement, the soft tissue defect was created around the knee joint. The defect center was above the patellar center (Figure 7A). Therefore, we selected algorithmic option No. 1, and the Cen-ALTP flap (16 × 14 cm^2^) was transferred to the defect with the pedicle anastomosed to the DB-LCFA (Figures 7B,C). The postoperative course was uneventful, and the transferred ALTP flap completely covered the soft tissue defect (Figure 7D).

### 4.2. Case 2 (patient No. 3)

**Figure 8:**
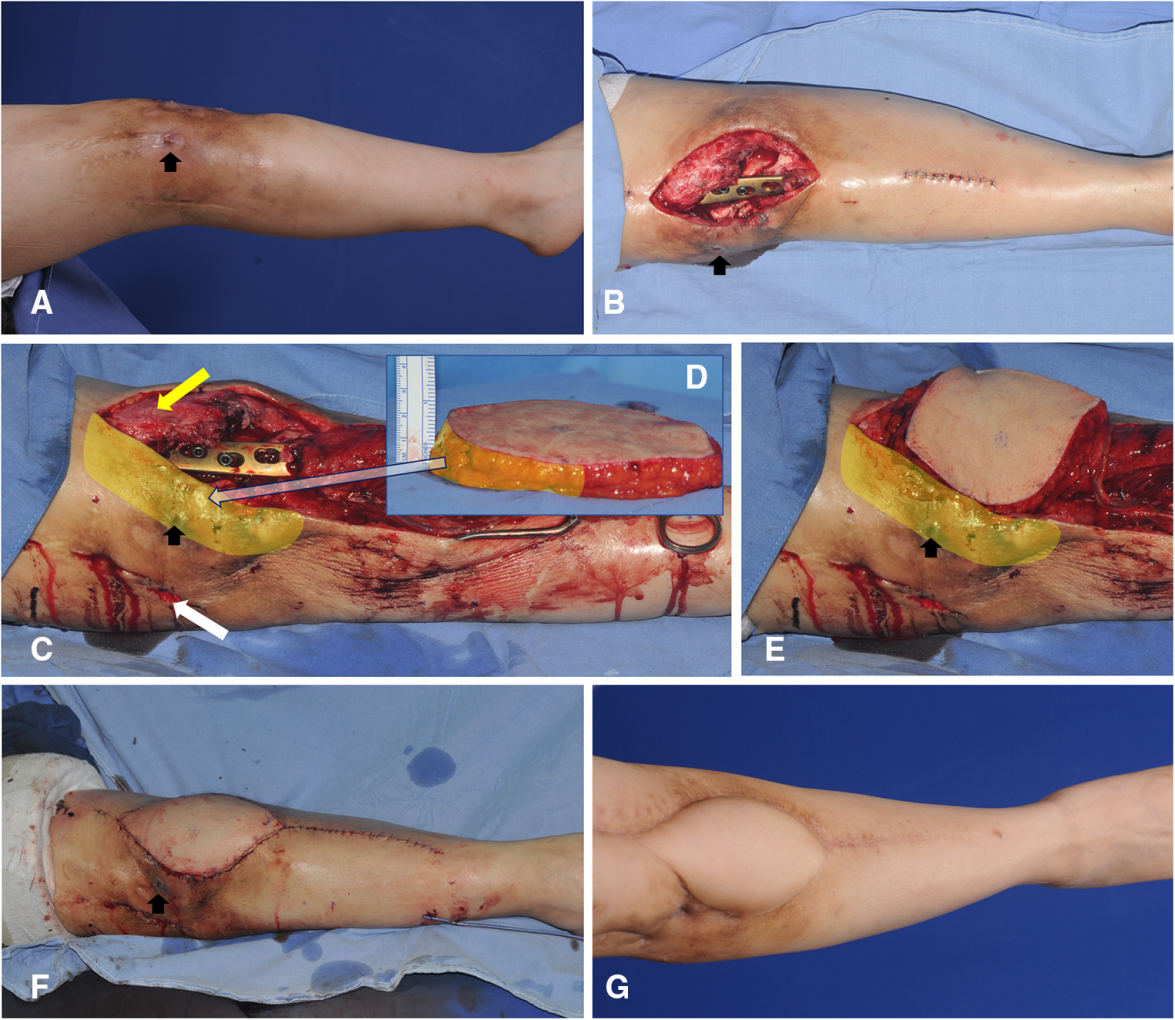
A clinical case demonstrating free transfer of Cen-ALTP flap using ATA and its concomitant veins as recipient vessels for the soft-tissue reconstruction around the knee (case No.3; Algorithmic option No.4; Black arrow indicates the sinus tract which communicates between underlying infected bones by osteomyelitis and overlying skin) (**A**) Damaged soft-tissue around knee joint was observed in a 49-year-old female patient suffering from chronic osteomyelitis. (**B**) The soft-tissue defect was created after radical debridement. The defect size was relatively small. (**C,D**) Orthopedic surgeons performed bony debridement through anterior (Yellow arrow) and lateral (White arrow) incisions. Plastic surgeons debrided damaged soft tissues around the anterior incision and preserved soft tissues between anterior and lateral incisions to limit the defect size to the optimal size range to be reconstructed safely by free ALTP flap. Alternatively, the space between infected bones and overlying soft tissues between anterior and lateral incisions was thoroughly cleaned up and occupied by healthy soft tissues of the transferred ALTP flap. (**E,F**) Cen-ALTP flap with a small round skin paddle (15 × 12 cm^2^) was harvested and transferred with the flap pedicle and was anastomosed to ATA and its concomitant veins. (**G**) Result 4 months postoperatively. (ALTP, anterolateral thigh perforator; Cen-ALTP flap, ALTP flap with centrally located perforators; ATA, anterior tibial artery).

A 49-year-old woman presented with chronic osteomyelitis of the knee (Figure 8A). Following debridement, the center of the created defect was below the patellar center (Figure 8B). Thus, we selected algorithmic option No. 4, and the Cen-ALTP flap (15 × 12 cm^2^) was transferred to the defect, with the flap pedicle anastomosed to the ATA (Figure 8C). The flap survived without complications (Figure 8D).

### 4.3. Case 3 (patient No. 6)

**Figure 9:**
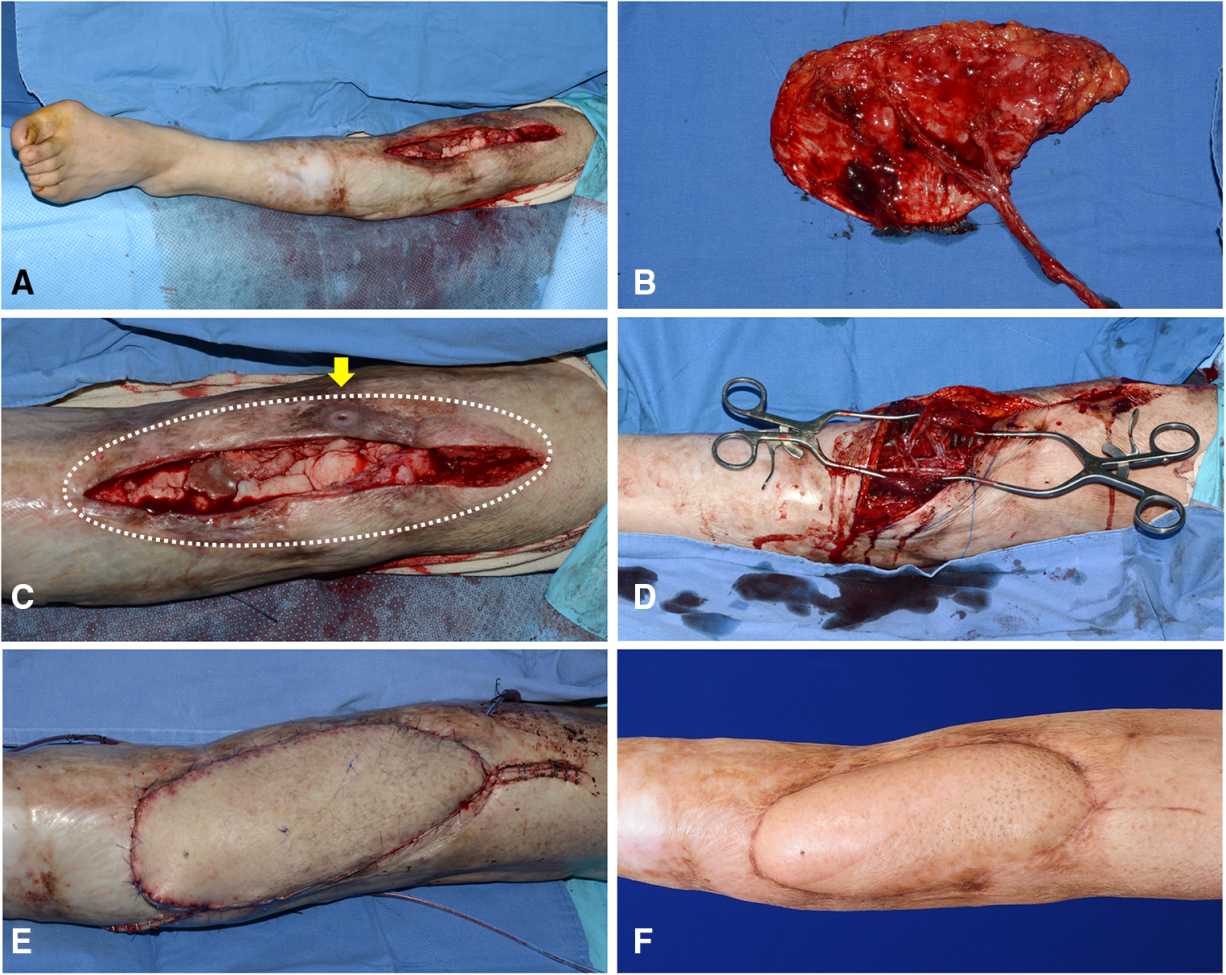
A clinical case presenting the reconstruction of the soft-tissue defect around the knee using Cen-ALTP flap, which is anastomosed to ATA and its venae comitantes (Case No.6; Algorithmic option No.4) (**A**) A wide soft-tissue defect was created by radical debridement in a 50-year-old male patient with chronic osteomyelitis. (**B**) Because the defect size was relatively large, Cen-ALTP flap with large long skin paddle (21 × 10 cm^2^) was harvested. (**C**) The wide damaged soft tissues (White dotted line), including the sinus tract, which communicates between underlying infected bones and skin (Yellow arrow), was excised. (**D**) The flap pedicle was anastomosed to ATA and its concomitant veins. (**E**) Immediate postoperative photography showed that a healthy free ALTP flap replaced previous unhealthy soft tissues overlying knee osteomyelitis. (**F**) Result 6 months postoperatively. (ALTP, anterolateral thigh perforator; Cen-ALTP flap, ALTP flap with centrally located perforators; ATA, anterior tibial artery).

A 50-year-old man presented with chronic post-traumatic osteomyelitis of the left knee joint. After thorough debridement, a long soft tissue defect was created. The defect center was below the patellar center (Figure 9A). For reconstruction, we selected algorithmic option No. 4, and the Cen-ALTP flap with a large long skin paddle (21 × 10 cm^2^) was transferred to the defect with the pedicle anastomosed to the ATA (Figures 9B,C). The flap completely survived and provided durable coverage to the knee joint (Figure 9D).

### 4.4. Case 4 (patient No. 2)

**Figure 10:**
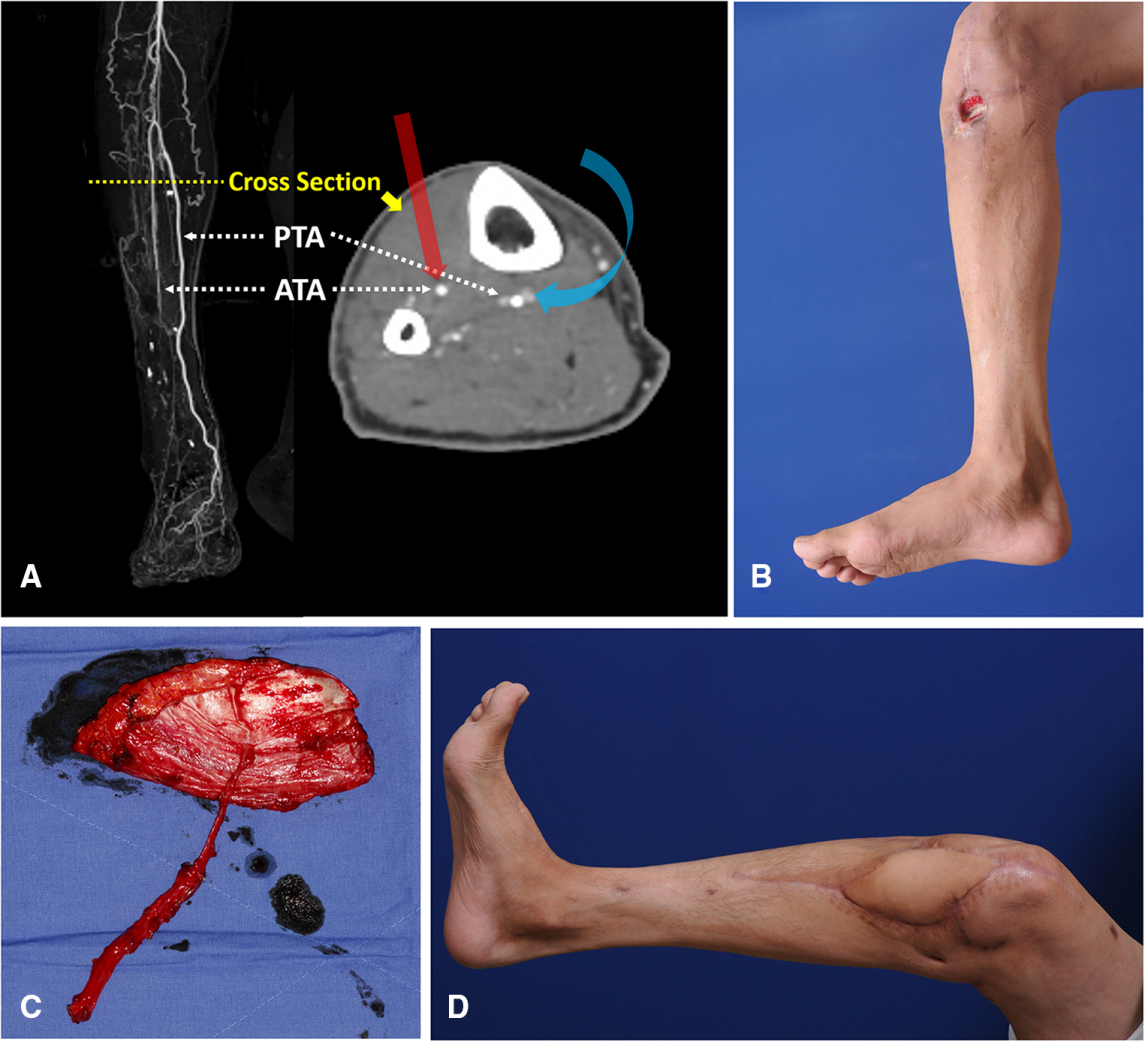
Reconstruction of soft-tissue defect around the knee by free transfer of Cen-ALTP flap using PTA and its concomitant veins as recipient vessels (Case No.2; Algorithmic option No.5) (**A**) Generally, when the defect center was below the patella center, ATA was first considered a recipient artery for the free flap coverage of the defect because of its ease of accessibility compared to PTA. Because PTA is located posterior to the tibia bone in the deep posterior muscle compartment, the approach to PTA in the proximal third of the calf is very hard. However, in cases where ATA is unavailable to use due to traumatic injuries or limited accessibility, PTA could be alternatively used as recipient vessel, just like in this case (The red arrow indicates straightforward access to ATA; the Blue arrow indicates tricky access to PTA). (**B**) A chronic wound was observed in a 46-year-old male patient with osteomyelitis of the knee joint. The wound center was below the patella center, and the proximal end of the defect after debridement was presumed to be below the patella center. (**C**) Because the defect size was relatively small, Cen-ALTP flap with small round skin paddle (13 × 10 cm^2^) was harvested. To achieve tension-free anastomosis, a long pedicle was included the flap. The flap pedicle was anastomosed to PTA and its concomitant veins without vein graft. (**D**) Result 5 months postoperatively. (Cen-ALTP flap, anterolateral thigh perforator flap with centrally located perforators; PTA, posterior tibial artery; ATA, anterior tibial artery).

The knee joint area was affected by chronic post-traumatic osteomyelitis in a 46-year-old man. Computed tomography angiography revealed discontinuity of blood flow in the ATA (Figure 10A). Following debridement, the defect center was speculated to be below the patellar center, and the whole defect was presumed to be below the patellar center (Figure 10B). Therefore, we selected algorithmic option No. 5. The Cen-ALTP flap (13 × 10 cm^2^) was then transferred with the pedicle anastomosed to the PTA (Figure 10C). The defect was successfully reconstructed (Figure 10D).

### 4.5. Case 5 (patient No. 13)

**Figure 11:**
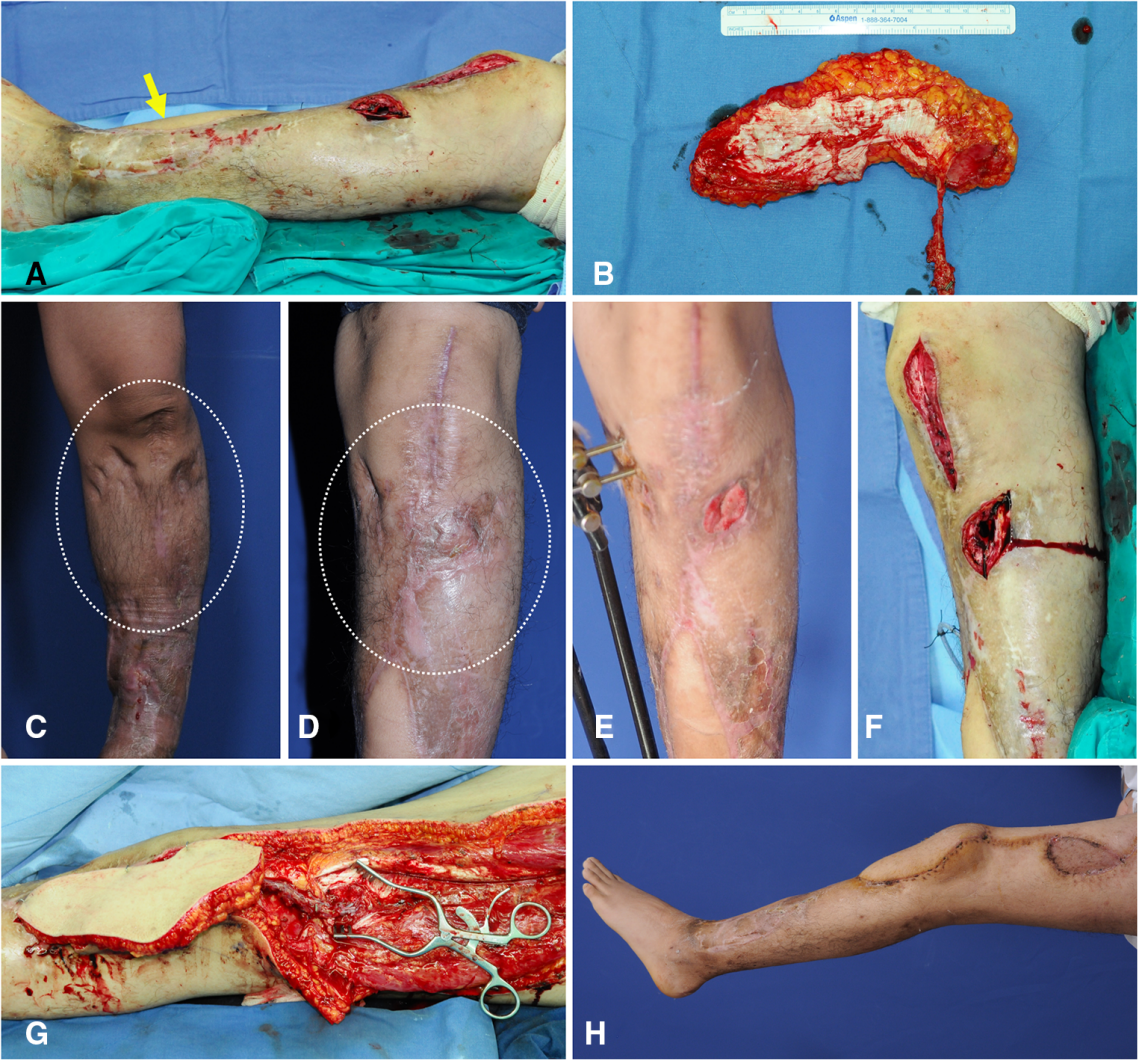
A clinical case showing free transfer of Ecc-ALTP flap using DB-LCFA and its concomitant veins as recipient vessels for the replacement of injured soft tissues by chronic osteomyelitis. (Case No.13; Algorithmic option No.7) (**A**) Radical debridement of the recurring osteomyelitis-related wound resulted in a soft-tissue defect with a long dimension in a 24-year-old male patient. The defect center was below the patella center. However, the defect extended into upper half of the patella. The yellow arrow indicates the thoracodorsal artery perforator flap, which was previously performed to reconstruct the osteomyelitis-related defect. (**B**) Since ATA was dissected and used as a recipient vessel in previous flap surgery, LCFA and its concomitant veins were selected as recipient vessels. Then, to provide adequate soft-tissue coverage to the area around the proximal tibia far from the expected vascular anastomosis site to DB-LCFA, an ALTP flap with large long skin paddle (21 × 11 cm^2^) supplied by an eccentrically located perforator was harvested. (**C,D**) Before the occurrence of the osteomyelitis-related wound on the knee region, the soft tissues around the knee region were already transformed into severely scarred tissues with poor vascularity due to repeated bone surgeries and surgical debridement procedures (White dotted circle). (**E**) When the wound caused by osteomyelitis was created, it was surrounded by multiple incisional scars and hard tissues. (**F**) Orthopedic surgeons debrided infected bone and soft tissues. (**G**) After radical excision of scarred soft tissues by plastic surgeons, the flap pedicle was anastomosed to DB-LCFA and its concomitant veins. After additional debridement of unhealthy soft tissues, the transferred ALTP flap was secured to adjacent healthy skin. (**H**) Result 2 months postoperatively. (Ecc-ALTP flap, anterolateral thigh perforator flap with eccentrically located perforators; DB-LCFA, descending branch of the lateral circumflex femoral artery; ATA, anterior tibial artery) Table (next page).

A 24-year-old man presented with recurring osteomyelitis of the left knee area. He had already undergone microsurgical reconstruction of an osteomyelitis soft-tissue defect using a thoracodorsal artery perforator flap, with the flap pedicle anastomosed to the ATA. After radical debridement, the center of the created defect was below the patellar center, and the superior portion of the defect extended to upper half of the patella (Figure 11A). Thus, we selected algorithmic option No.7, and the Ecc-ALTP flap with long large skin paddle (21 × 11 cm^2^) was transferred with the flap pedicle anastomosed to the DB-LCFA (Figures 11B,C). Successful coverage was achieved without complications (Figure 11D).

## 5. Discussion

Soft tissue defects occurring around the knee are common and can result from traumatic injuries (14), oncologic resection of soft tissue or bone tumors (15), and surgical release of burn scar contracture (16). In addition, these defects can be created by wound infection and multiple surgical procedures. Wound problems after total knee arthroplasty are a typical example. These problems may affect up to 20% of patients who undertook total knee arthroplasty and cause soft tissue necrosis and implant exposure. Severe infections may result in prosthesis failure or limb loss (17). Repeated surgical debridement could be performed to manage these infectious wounds, but they might also lead to soft tissue breakdown that needs to be reconstructed (17–19). Similarly, in patients with knee osteomyelitis, the soft tissues around the knee are usually affected by the accumulated damage caused by the infection itself and by therapeutic trials, including repeated debridement and irrigation. Finally, damaged soft tissues around the knee are radically removed to cure osteomyelitis with the aim of reconstruction. However, there have been no reports of reconstructive trials of these defects. This probably implies that the reconstructive procedures for these defects are not popularly tried due to the uncertainty about the availability of local tissues or potential recipient vessels.

Optimal reconstruction of knee defects is challenging for plastic surgeons because thin and elastic coverage should be restored to facilitate wound healing and any concurrent orthopedic operation (10, 14). Thus, surgeons should try to find the best surgical options by assessing the location and size of the defect, previous operation history, and infection status. In patients with relatively small and shallow knee defects, free from infection and bone exposure, most defects can be managed with local tissues such as primary closure, local advancement flaps, and skin grafting (5, 20). However, more complex and invasive flap surgeries should be performed for more extensive and profound defects. For this purpose, a wide variety of flaps such as local random or axial pattern fasciocutaneous flaps, local transposition muscle flaps, perforator-based propeller flaps, and microsurgical free flaps have been used (21–28). Nevertheless, free tissue transfer is preferred to reconstruct complex soft tissue defects around the knee because local tissue options, such as muscle flaps or rotational fasciocutaneous flaps are usually unreliable due to scarring and poor blood supply caused by traumatic injuries and repeated surgeries (29). This preference becomes more apparent when dealing with infectious soft tissue defects already treated with multiple surgeries. Because these defects and their surrounding damaged areas are required to be replaced by healthy distant soft tissues to promote infection control and wound healing, free tissue transfer can be an ideal option to satisfy this requirement (30). In addition, skin paddles of free flaps can be tailored to suit the diverse shapes and sizes of each defect, while also delivering various types of deep tissues such as fascia, tendon, muscle, and bone to satisfy the particular necessity of defects (31). In recent years, perforator flaps have tended to substitute muscle flaps in almost all free tissue transfers because they provide not only similar functional benefits on the coverage of deep tissues and infection control, but also extended usability based on the wide dimension of skin paddles and long pedicles. The advent of perforator flaps has opened a new era in lower extremity reconstruction. Almost all defects in the lower limb have been reconstructed using perforator flaps, and the knee lesions are no exception. The ALTP flap was most commonly used, followed by the thoracodorsal artery perforator (TDAP) flap, superficial iliac circumflex perforator (SCIP) flap (32), medial sural artery (MSA) perforator free flap (33), and deep inferior epigastric perforator flap (34). Perforator flaps have advantages in tension-free pedicle anastomosis due to adequately long pedicle length and minimization of donor site morbidity, by not including muscle in the flap (35).

The ALTP flap is ideal to cover osteomyelitis-related knee defects because it can present an adequate size of pliable soft tissue to enable easier revisional surgeries (36). They can be utilized as perforator-based pedicle flaps. Wong et al. reported a flap success rate of 100% in a series of eight patients treated with anterolateral thigh flaps (six pedicled flaps and two free flaps) for soft tissue restoration of the knee. They suggested employing the anterolateral thigh flap as an antegrade advancement flap for minor abnormalities at or above the patella, and either a distally based propeller or free flap for larger and more inferior knee deformities (37). However, these pedicled ALTP flaps are not appropriate for the reconstruction of osteomyelitis-related knee defects. The final defect following radical debridements is usually too large to be reconstructed using an antegrade advanced ALTP flap. In addition, the genicular arterial network might be damaged due to chronic infectious inflammation and repeated surgical trauma; therefore, it probably could not guarantee adequate blood supply to the distally based propeller ALTP flap. According to the shape, size, location, and depth of the soft-tissue defects resulting from infection and related management, normal vascular anatomy is likely distorted, and available surgical options may be changed (8). Other local reconstruction options have also been unavailable for similar reasons.

Therefore, we performed only free ALTP flap transfer to reconstruct osteomyelitis-related soft tissue defects of the knee. Many surgeons have used a free ALTP flap for soft tissue reconstruction of the knee region, and successful results have been obtained. Lee et al. reported a successful limb salvage rate (82%) at a mean of 48-month follow-up after free ALTP flap (*n* = 22) and free LD flap (*n* = 1) transfer to the infected knee area following total knee arthroplasty in patients who underwent multiple previous surgeries. They suggested that free ALTP flap transfer can provide reliable soft tissue coverage of infected knee wounds (38). In this study, we also achieved excellent limb salvage results (100%) at a mean of 27-month follow-up. Although orthopedic bone debridement and reinsertion of antibiotic-loaded bone cement were additionally performed in some patients after reconstruction, the transferred ALTP flap could provide durable and flexible coverage to the knee joint, allowing repeated surgeries and daily knee joint motion. Unlike previous studies, we have tried to optimize the shape and size of the ALTP flap depending on the location, the distance from the provisional vascular anastomosis site, and the size of each soft-tissue defect around the knee. Therefore, to cope with diverse defects and minimize postoperative circulatory problems, we have used two types of ALT flaps: Cen-ALTP flap and Ecc-ALTP types. Furthermore, we individualized the shape of the skin paddle in each ALTP flap according to the requirement for the reconstruction.

The knee region has a complicated vascular network that mainly consists of the popliteal artery and its branches, including the sural arteries, superior or inferior genicular arteries (GAs), and ATA or PTA. Additionally, the descending GAs, which arise from the femoral artery, and DB-LCFA, which arise from the profunda femoris artery, were also connected to this network (39, 40). Some authors have reported that perforating or muscular branches of the profunda femoris (deep femoral) artery could be used as a recipient artery for free soft tissue transfer to the knee joint area (5) or free vascularized bone graft to the femoral head (41) or shaft (42). The profunda femoris artery arises from the femoral artery and runs down the thigh deep to the adductor longus muscle. It gives off three perforating branches and continues to the distal terminal segment, and the mean diameters of these branches are similar to the diameters of DB-LCFA or peroneal artery (42). Thus, these sizable branches, which are well protected by muscles, are also can be a good candidate for recipient vessels. Almost all of these vessels around the knee can be used as recipient vessels, and each vessel has its own set of merits and drawbacks. Despite these various choices of recipient vessels, controversy regarding the best vessel for a free-flap surgery around the knee region still exists. Therefore, reconstructive surgeons have usually tried to select the best available vessel based on each patient's anatomy on a case-by-case basis. We also attempted to identify a reliable candidate for recipient vessels to reconstruct osteomyelitis-related knee defects. We realized that there is a paucity of available recipient vessels in patients with chronic post-traumatic osteomyelitis of the knee joint because almost all small vessels in the genicular arterial system were damaged or destroyed during the management of osteomyelitis. In contrast, we found that DB-LCFA or tibial vessels (ATA and PTA) could serve as valuable recipient vessels because they are generally well preserved by the protection of bulky muscles (8). PTA is more trustworthy as a recipient artery because the ATA has a substantially greater rate of injury and severe damage than the PTA, after lower extremity trauma (43). However, in our experience, the severity of the damage following trauma was not notably different between ATA and PTA. Conversely, the PTA was not useful as a recipient artery because of its inaccessibility due to its deep location, just posterior to the tibia, under the thick calf muscles. As a conclusion, we used the most appropriate vessel among ATA, PTA, and DB-LCFA as recipient vessels for free ALTP flap transfer, considering each patient's clinical features.

We established an algorithm to select the optimal ALTP flap type and recipient vessels in various clinical situations. During the development of this algorithm, we attempted to encompass almost all possible situations and build a tailored countermeasure suitable for each situation. As a result, we produced seven surgical options consisting of the most appropriate recipient vessels and the optimal flap type. In this study, we reviewed the surgical outcomes based on the surgical decision using this algorithm and verified its reliability and usefulness. In the future, we plan to modify and upgrade the algorithm by considering the accumulated surgical results. Furthermore, we are scheduled to conduct a prospective, multi-center, comparative study to compare the surgical outcomes between algorithmic and intuitive approach.

## 6 Conclusion

The authors developed an algorithm to provide surgical options for optimal recipient vessels and an ALTP flap type for free tissue transfer to osteomyelitis-related soft-tissue defects around the knee. We then performed individualized microsurgical reconstructions based on a selected option according to the defect size and location in each patient and achieved sufficient successful coverage of the knee defect to enhance eradication of infection and protection from it. We believe that our algorithm could help surgeons devise a strategic reconstructive plan for peri-knee soft tissue defects and obtain successful surgical outcomes in patients with chronic post-traumatic osteomyelitis of the knee.

## Data Availability

The original contributions presented in the study are included in the article/Supplementary Material, further inquiries can be directed to the corresponding author.
